# Cu-Ag nanoparticles positively modulating the endophytic bacterial community in tomato roots affected by bacterial wilt

**DOI:** 10.3389/fmicb.2025.1579517

**Published:** 2025-07-16

**Authors:** Weimin Ning, Xuefeng Bao, Lei Jiang, Mei Yang, Tianhao Lei, Maoyan Liu, Yong Liu

**Affiliations:** ^1^School of Agricultural Science, Xichang University, Xichang, China; ^2^Key Laboratory of Pest Management of Horticultural Crop of Hunan Province, Hunan Academy of Agricultural Science, Changsha, China; ^3^Sichuan Provincial Key Laboratory for Research and Utilization of Panxi Special Crops, Xichang University, Xichang, China

**Keywords:** tomato, *Ralstonia solanacearum*, Cu-Ag nanoparticles, endophyte, bacterial community

## Abstract

**Introduction:**

Tomato bacterial wilt (TBW) is a destructive soil-borne bacterial infection caused by Ralstonia solanacearum. Various nanoparticles have been employed as antibacterial agents to manage TBW via soil application. However, research on the effects of nanoparticles on plant endophytes remains limited.

**Methods:**

Here, an analysis of the endophytic bacterial community was performed on healthy and infected tomatoes that were treated with Cu-Ag nanoparticles and thiodiazole-copper via high-throughput 16S rRNA gene amplicon sequencing.

**Results:**

The relative abundance levels of beneficial bacteria, including Acidobacteriota, Firmicutes, Actinobacteriota, and Myxococcota, were higher in infected tomato roots treated with Cu-Ag nanoparticles compared with thiodiazole-copper. Functional predictions show that Cu-Ag nanoparticles may affect pyruvate metabolism, oxidative phosphorylation, purine metabolism, carbon metabolism, secondary metabolite production, and the metabolic pathways associated with microbial communities.

**Discussion:**

These results could reveal the mechanism by which nanoparticles influence the endophytic microbiomes of plant roots and direct the rational application of nanoparticlesin sustainable agriculture.

## Introduction

Endophytes are non-pathogenic microorganisms that inhabit plant tissues and can be isolated from the surface or extracted from inside the plants; they have a crucial role in promoting plant growth (Wang et al., 2022; Ali et al., 2024; Agnusdei et al., 2024). Endophytes exhibit phenotypic variety and can be classified as obligatory or facultative endophytes according to their connection with host plants (Glynou et al., 2018; Pinto-Carbó et al., 2018). In addition, they may be classified as transitory or true endophytes based on their transmission mode (Afzal et al., 2019). Endophyte transmission mechanisms include vertical, horizontal, and mixed-mode transmission (Rufián et al., 2022). Vertical spread occurs through seeds or pollen (Wang et al., 2022), while horizontal spread occurs via soil composition, air conditions, insect activity, and other environmental variables (Abdelhamid et al., 2024). Certain endophytes spread through both vertical and horizontal transmission pathways also referred to as mixed-mode transmission (Ayesha et al., 2021; Groben et al., 2024). The soil-to-root transmission route is the most thoroughly studied among the various transmission pathways (He et al., 2023; Xing et al., 2023).

Endophytic colonization is regulated by genetic, metabolic, or growth regulator factors (Hardoim et al., 2015; MartínezMedina et al., 2017). Endophytes are vital to host adaptability and nutrition supply, and they mediate complex and multifaceted protective mechanisms (Gao et al., 2021; Pal et al., 2022; Shah et al., 2023). Certain endophytes can solubilize iron in soil to increase plant uptake and promote the synthesis of vital nutrients, as well as the expression of genes associated with micronutrient production (Bharadwaj et al., 2012; Shu et al., 2023; Waqas et al., 2017). Certain endophytes may produce auxins, phosphates, gibberellins, cytokinins, and ethylene that can improve plant cell elongation and proliferation (Burragoni and Jeon, 2021; Narayanan et al., 2022). Numerous endophytes can strengthen plant defense systems against pathogens by inducing systemically acquired resistance and by regulating hypersensitive responses (Singh et al., 2022). In addition, endophytes can defend against necrotrophic diseases by stimulating the generation of lipopeptides, cyclic cationic lipopeptides, and other secondary metabolites (Marokane-Radebe et al., 2024; Velmurugan et al., 2023). Furthermore, endophytes can enhance both antibiotic activity and antagonistic effects (Singh and Shyu, 2024). Overall, endophytes perform vital functions in protecting plants against pathogens.

Soil-borne plant diseases can markedly decrease agricultural crop yields globally, resulting in considerable economic losses (Greff et al., 2023; Harish, 2022). *Ralstonia solanacearum* (*R. solanacearum*) is Gram-negative, soil-borne bacteria that causes bacterial wilt (BW) (Manickam et al., 2021). BW is the second most damaging bacterial disease worldwide, affecting both the quality and yield of key agricultural crops such as tomato, pepper, potato, and ginger (Yuliar et al., 2015). Nanoparticles have emerged as an efficient strategy to manage BW because of their small size, high surface area to volume ratio, and superior penetration capacities (Khairy et al., 2022; Gul et al., 2024; Khan et al., 2021; Guo et al., 2022). Therefore, nanoparticles could serve as carrier molecules for pesticides, fertilizers, and antimicrobials, enabling the timely and targeted release of active substances to improve their effectiveness (Liang et al., 2022; Wang et al., 2023).

Nanomaterials can influence the composition of host plant endophyte microbiomes via multiple pathways after being applied to plants (Eid et al., 2021; Meena et al., 2021; Parkinson et al., 2022). Zhang et al. (2024) highlighted that zinc oxide nanoparticles enhance soybean tolerance to *aluminum* toxicity, resulting in an increased diversity of root endophytic microorganisms and an increase in the populations of *Aureimonas, Luteimonas,* and *Sphingomonas*. Love and Hemalatha (2023) found that silver (Ag) nanoparticles biosynthesized by *Cronobacter sakazakii* can be utilized as fungicides to manage the *Mucor racemosus* in crops. Shang et al. (2024) investigated that selenium nanoparticles could restore the composition of the rice endophyte community and substantially increase the relative abundance of the beneficial species *Azospirillum*. Thus, the effects of various nanomaterials on plant endophytes are distinct.

To perform a thorough investigation of the nanoparticles influencing the plant endophyte bacterial community. Cu-Ag nanoparticles were applied to healthy tomato plants and plants infected by *R. solanacearum*. Cu-Ag nanoparticles had a small size and displayed effectiveness in protecting tomatoes against *R. solanacearum* in previous study (Ning et al., 2024). High-throughput 16S rRNA gene amplicon sequencing was utilized to study potential alterations in the tomato root endophyte microbiome. The bioaccumulation of Ag, copper (Cu), phosphorus (P), and molybdenum (Mo) in tomato roots was measured using inductively coupled plasma mass spectrometry (ICP-MS). This study revealed the influence of Cu-Ag nanoparticles on the microbial community of tomato root endophytes and enhanced understanding of the disease prevention and control mechanisms underlying from the perspective of nanoparticle’s effects on the microbial community.

## Materials and methods

### Materials

Nitric acid (HNO₃) was purchased from Aladdin Biochemical Technology Co., Ltd., Shanghai, China; thiodiazole-copper was purchased from Longwan Chemicals Co., Ltd., Zhejiang, China; Tomato seeds (cv. zuanhongmeina) and *Ralstonia solanacearum* were stored at the Key Laboratory of Pest Management of Horticultural Crop of Hunan Province, Hunan Academy of Agricultural Science; the MagPure Soil DNA LQ Kit was purchased from Magen Biotechnology Co., Ltd., Guangzhou, China; the Qubit dsDNA Assay Kit was purchased from Life Technologies Pvt. Ltd., California, America; Tks Gflex™ DNA Polymerase was purchased from Takara Biomedical Technology Co., Ltd., Beijing, China; the synthesis of Cu-Ag nanoparticles was based on a previous study (Ning et al., 2024).

### *Ralstonia solanacearum* culturing

*Ralstonia solanacearum* was grown on nutrient broth comprising 3.0 g/L beef extract, 1.0 g/L yeast extract, 5.0 g/L peptone, and 10.0 g/L glucose at pH 7.0 and cultured in a bacteriological incubator maintained at 37°C. The bacterial cultures were collected at the mid-exponential growth phase and centrifuged at 3000 rpm for 5 min at 4°C to collect the cells. Subsequently, the bacterial pellet was washed three times with deionized water to remove the medium. Then, the cells were resuspended in deionized water, diluted to OD_600_ = 0.1, and were used in the following experiments.

### Plant growth and nanoparticle exposure

Tomato seeds were washed thoroughly with a 10% sodium hypochlorite solution, were washed with deionized water, and allowed to sprout on a wet dish until two or three true leaves had grown. Subsequently, individual tomato seedlings were transferred into individual plastic pots, and the plants were transferred into a greenhouse environment maintained at a temperature of 27 ± 2°C, a relative humidity of 60%, and 16 h of daylight. After 30 days of growth, the tomato seedlings showing uniform growth were randomly divided into six groups, including tomato solely infected with *R. solanacearum* (G6); infected tomato treated with Cu-Ag nanoparticles (G7); infected tomato treated with thiodiazole-copper (G8); healthy control tomato (G9); healthy tomato treated with Cu-Ag nanoparticles (G10); and healthy tomato treated with thiodiazole-copper (G11). Each treatment comprised six tomato plants and was replicated three times. Thiodiazole-copper was diluted 500-fold according to the instructions, and Cu-Ag nanoparticles were diluted to 20 μL/mL based on previous studies for the following experiments. Freshly prepared *R. solanacearum* solution (15 mL; OD_600_ = 0.1) was added to the soil of the treatments G6, G7, and G8. After 48 h, G6 was supplemented with 20 mL sterile water; G7 was supplemented with 20 mL Cu-Ag nanoparticles; G8 was supplemented with 20 mL thiodiazole-Cu. Meanwhile, G9, G10, and G11 only were 20 mL sterile water, 20 mL Cu-Ag nanoparticles, and 20 mL thiodiazole-copper applied, respectively. All tomato plants were grown in a greenhouse, and the greenhouse conditions were kept constant as previous.

### Roots sample harvesting procedures

The tomato roots were harvested 15 days after the applying Cu-Ag nanoparticles and thiodiazole-copper. The tomato root systems were removed from the containers, and the bulk soil discarded via gentle shaking. The roots were transferred into sterile tubes supplied with 30 mL of phosphate-buffered saline (PBS) buffer (pH 7.0). The tubes were shaken carefully to remove as much soil as possible from the roots; then, sterile tweezers were used to transfer the roots to a new sterile tube containing PBS. This step was repeated twice at 20 min per separation. The root samples were collected in new tubes, immediately frozen in liquid nitrogen, and stored at −80°C until DNA extraction.

### Measurement of element contents

The concentrations of Ag, Cu, P, and Mo in the root tissues were measured. The root tissues were oven-dried at 65°C for 3 days. Afterward, they were pulverized into a fine powder using an electric blender. This powder was transferred into digestion vials to soak for 1 h in a solution of HNO₃ and H₂O₂ (v/v = 1:4). After digestion, sterile water was added to the digested roots to attain a final volume of 50 mL. The Ag, Cu, Mn, and Mo contents were determined using ICP-MS (NexlON 1000G, Massachusetts, USA). The P content was measured using inductively coupled plasma-optical emission spectroscopy (ICP-OES; Agilent Technologies 5,110, California, USA).

### Roots DNA extraction and sequence amplification

Bacterial DNA was extracted from tomato samples using a DNeasy PowerSoil kit (Qiagen N. V Co., Ltd., Hilden, Germany) following the manufacturer’s instructions, and DNA quality and concentration of each root sample were evaluated using agarose gel electrophoresis (1% in TAE buffer) and a Nanodrop 2000 spectrophotometer (Thermo Fisher Scientific Lnc., Waltham, USA). The high-quality DNA samples were selected for subsequent polymerase chain reaction (PCR) amplification; the V3-V4 region of the bacterial 16S ribosomal RNA gene was amplified using universal primer pairs 343F and 798R (343F: 5′-TACGGRAGGCAGCAG-3′; 798R: 5′-AGGGTATCTAATCCT-3′) (Nossa et al., 2010). The PCR reactions were conducted in 30 μL aliquots. The reaction mix contained 15 μL of 2 × Gflex PCR Buffer, 1 μL of 5 pmol/μl forward primers, 1 μL of 5 pmol/μl reverse primers, 0.6 μL of 1.25 U/μl Tks Gflex DNA Polymerase, 1 μL of template DNA, and 11.4 μL of H₂O. The presence of the expected amplification product was checked using gel electrophoresis, and the PCR products were purified using Agencourt AMPure XP beads (Beckman Coulter, Inc. California, USA) and quantified using a Qubit dsDNA assay kit (Life Technologies Pvt. Ltd., California, USA). Finally, the library was sequenced on the Illumina NovaSeq6000 (Illumina, Inc., California, USA) with 2 × 250 paired-end mode.

### Statistical analysis

Raw sequencing data were obtained in the FASTQ format. The cutadapt function of QIIME2 software (version 2023.09, Northern Arizona University, USA.) was used to detect paired-end reads and remove adapters. After trimming, the paired-end reads were filtered for low-quality sequences, denoising, merging, and the detection and removal of chimeric reads using the default settings in QIIME2 software (Erdenebileg et al., 2024). Finally, the program produced representative readings and an amplicon sequence variant (ASV) abundance table. The ASV table was applied to establish the Chao1 and Shannon diversity via QIIME2 software (Miaow et al., 2021). The unweighted UniFrac distance matrix was calculated employing the “vegdist” function in the free software computing environment R package (version 3.5.1). Subsequently, a principal coordinate analysis (PCoA), based on the Bray–Curtis distance, as well as nonmetric multidimensional scaling (NMDS), were performed using the R package (3.5.1) (Jing et al., 2024). The “ggplot2” based R package (3.5.1) was utilized to generate the abundance patterns of microbial community composition at the phylum, class, and genus levels, as well as to determine significant differences among bacterial communities in various groups using a one-way analysis of variance (ANOVA) and the Kruskal-Wallis test at a confidence level of 95%. For statistical analyses, the analysis of similarity (ANOSIM) and permutational multivariate analyses of variance (PERMANOVA) were performed (Wen et al., 2023). Moreover, the linear discriminant analysis (LDA) effect size (LEfSe) based on Kruskal-Wallis and pairwise Wilcoxon tests was applied by using the default parameters to identify the most abundant microbial taxa present under different treatments (Gao et al., 2022). For the co-occurrence network evaluation, the tomato root bacterial correlations were calculated using Louvain methods based on the abundance of bacteria at the phylum level (Salnikov et al., 2018). Functional predictions for the microbial community were conducted utilizing PICRUSt2 software (Dalhousie University, Canada) with default analytic settings (Douglas et al., 2020). GraphPad Prism 10 (GraphPad Software, USA) and Origin 2024 (OriginLab, USA) were employed to construct graphs.

## Results

### Nanoparticles influenced the number of endophytic communities

**Figure 1:**
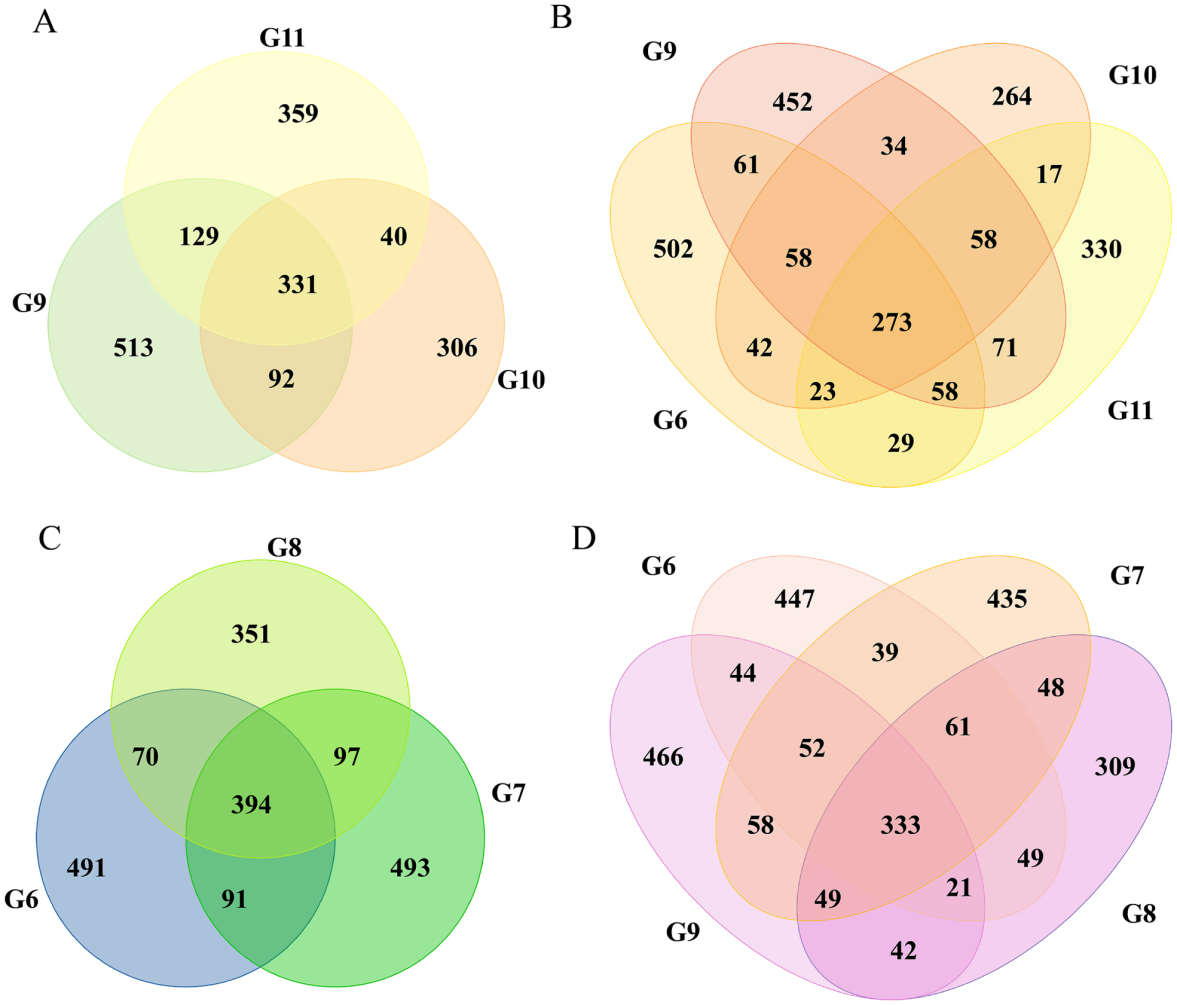
Venn diagram illustrating the shared and unique ASVs in different tomato roots. The shared and unique ASVs in groups G9, G10, and G11 **(A)**. Groups G6, G9, G10, and G11 **(B)**. Groups G6, G7, and G8 **(C)**. Groups G6, G7, G8, and G9 **(D)**.

The Venn diagrams were used to illustrate the microbial constituents of tomato roots under various treatments. The number of ASVs in each group was between 283 and 673; the distribution of six groups shared ASVs with 69 reads (Supplementary Figure S1); more ASVs were found in healthy (1065) than in infected tomato (1046). A comparison of the three healthy tomato found that 331 ASVs were shared, representing 12.3% of the total. There were 394 ASVs shared in three infected groups (Figures 1A,C). Additional analyses were conducted between healthy and infected tomato. The comparison of groups G10 and G11 showed that 40 out of 1,628 ASVs were shared, whereas 4.9% (97/1987) of ASVs were shared in groups G7 and G8 (Figures 1B,D). In summary, a greater number of intrinsic bacteria was maintained in infected tomato roots compared to healthy tomato, with the highest amounts observed in infected roots treated with nanoparticles.

### Cu-Ag nanoparticles affect the diversity of roots endophytic bacteria

**Figure 2:**
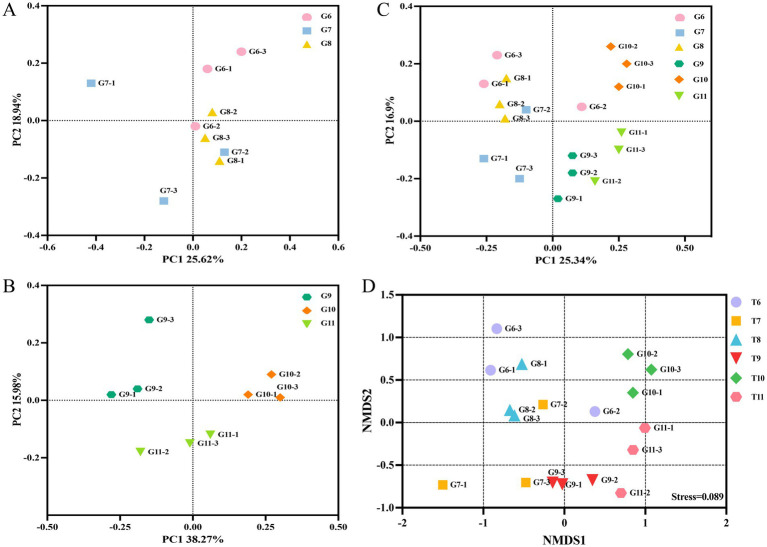
The beta-diversity of the bacterial community. Principal coordinate analysis (PCoA) of the bacterial community based on Bray-Curtis distance in the groups G6, G7 and G8 **(A)**. The PCoA of groups G9, G10 and G11 **(B)**. The PCoA of groups G6, G7, G8 G9, G10 and G11 **(C)**. Community clustering of bacteria between different groups was visualized in a two-dimensional NMDS plot **(D)**.

The influence of nanoparticles on the diversity of the endophytic microbial community in tomato roots was evaluated using alpha and beta diversity. The Chao1 and Shannon indices indicated no significant changes between the diseased tomato treated with nanoparticles; differences were observed between the healthy control tomato and the healthy tomato treated with nanoparticles (Supplementary Figure S2). The PCoA indicated that Principal Component 1 (PC1) accounted for 25.62% of the variance and Principal Component 2 (PC2) accounted for 18.94% of the variation in the community of diseased tomato roots (Figure 2A). In healthy tomato, PC1 accounted for 38.27% and PC2 accounted for 15.98% of the difference in bacterial communities (Figure 2B). The variances explained by PC1 and PC2 were 25.34 and 10.3% in all groups, respectively (Figure 2C). The two-dimensional NMDS plot revealed that tomatoes from the same treatment tended to cluster, with a stress value of 0.089 (Figure 2D). Overall, nanoparticles influenced the diversity of the endophytic microbial community in tomato roots.

### Nanoparticles increased the relative abundance of beneficial microbes

**Figure 3:**
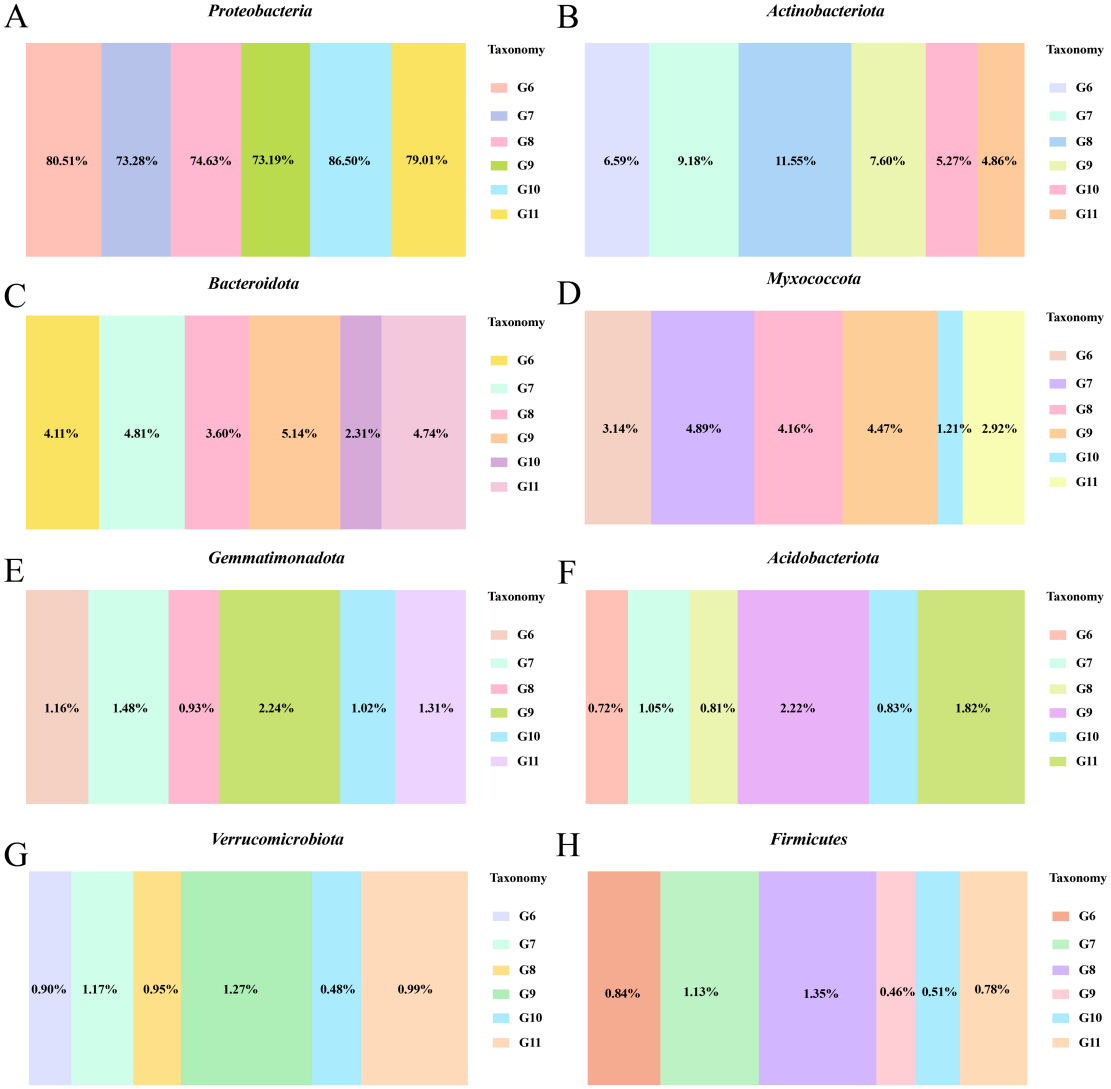
The bacterial community abundance at the phylum level of healthy tomato roots and infected tomato roots after different treatments. The relative abundance of *Proteobacteria* **(A)**, *Actinobacteriota* **(B)**, *Bacteroidota* **(C)**, *Myxococcot*a **(D)**, *Gemmatimonadota* **(E)**, *Acidobacteriota* **(F)**, *Verrucomicrobiota* **(G)**, and *Firmicutes* **(H)**.

The number of endophytic bacteria categorized at the phylum, class, order, family, genus, and species levels in tomato roots differed (Supplementary Figure S3). At the phylum level, Proteobacteria was the most abundant bacteria among the six treatment groups. The relative abundance of Proteobacteria was significantly higher in control diseased tomato (80.51%) than control healthy tomato (73.19%). The prevalence of *Proteobacteria* in diseased tomato treated with Cu-Ag nanoparticles (73.28%) was comparable with that in control healthy tomato (Figure 3A). The relative abundances of *Actinobacteriota, Bacteroidota,* and *Myxococcota* decreased in control infected tomato (6.59%, 4,11, and 3.14%) compared with control healthy tomato (7.60%, 5,14, and 4.47%); however, the numbers of both of these bacteria increased after applying Cu-Ag nanoparticles to infected tomato (9.18, 4.81, and 4.89%) (Figures 3B–D). The Cu-Ag nanoparticle raised the relative abundances of *Gemmatimonadota, Acidobacteriota, Verrucomicrobiota,* and *Firmicutes* in infected tomato (1.48, 1.05, 1.17, and 1.13%) in contrast with control infected tomato (1.16, 0.72, 0.90, and 0.84%) (Figures 3E–H).

The top five bacterial classes observed across all the tomato plant root samples included *Gammaproteobacteria*, *Alphaproteobacteria*, *Actinobacteria*, *Bacteroidia*, and *Polyangia*. The Cu-Ag nanoparticles reinstated the abundance of *Gammaproteobacteria*, *Actinobacteria*, *Bacteroidia* and *Polyangia* in diseased tomato, aligning them more closely with those in healthy control tomato. Cu-Ag nanoparticles and thiodiazole-copper reduced the amount of *Alphaproteobacteria* on both healthy and diseased tomato roots (Supplementary Figure S4A). In addition, the relative abundances of microbes at the order level were investigated. Nanoparticles reduced the amount of *Burkholderiales* (12.58%) and *Rhizobiales* (16.02%) in comparison to the control infected tomato roots, which exhibited levels of 23.77 and 21.14%, respectively. The invasion of *R. solanacearum* deecreased the relative abundances of *Xanthomonadales*, *Caulobacterales*, *Micrococcales*, *Streptomycetales,* and *Polyangiales* compared with healthy control tomatoes; whereas Cu-Ag nanoparticles augmented their numbers in diseased tomato roots (Supplementary Figures S4B,C). At the family level, Cu-Ag nanoparticles reduced the abundance of *Comamonadaceae*, *Rhizobiaceae*, *Oxalobacteraceae*, *Sphingomonadaceae*, and *Burkholderiaceae* in diseased tomato roots (Supplementary Figure S4D). In summary, the presence of Cu-Ag nanoparticles notably enhanced the relative abundance of certain bacteria, particularly in diseased tomato roots.

### LEfSe analysis of the endophytic bacteria

**Figure 4:**
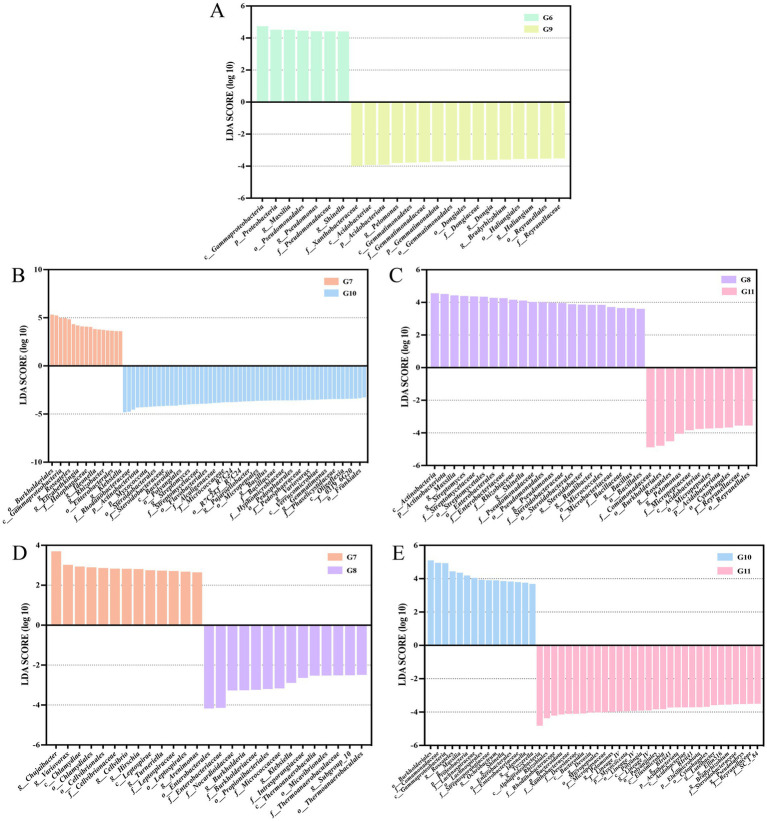
Linear discriminant analysis (LDA) effect size (LEfSe) shown the significantly different endophytic abundant taxa in the tomato roots. Histograms of LDA scores in groups G6 and G9 **(A)**. Groups G7 and G10 **(B)**. Groups G8 and G11 **(C)**. Groups G7 and G8 **(D)**. Groups G10 and G11 **(E)**. The figure only shows species with significant differences in LDA value (log 10) above 3.0.

To identify the potential biomarkers that exhibit the most significant variation in endophytic microbial communities among different conditions. LEfSe analysis was used to identify treatments with LDA scores greater than 3.0. Variations were observed in the microbial composition of endophytes in control healthy tomato roots and control diseased tomato roots. A total of 16 biomarkers were found in control healthy tomato. The amounts of seven bacteria, including class *Gammaproteobacteria*, phyla *Proteobacteria,* genera *Massilia, Pseudomonas*, and *Shinella*, order *Pseudomonadales*, and family *Pseudomonadaceae* were abundant in control infected tomato (Figure 4A). Healthy tomato roots treated with nanoparticles exhibited considerable enrichment of the order *Xanthomonadales*, family *Rhodanobacteraceae*, genera *Rhodanobacter*, phyla *Actinobacteriota* and *Myxococcota*, along with 53 other bacterial taxa were significantly enriched. Only 17 bacterial taxa were significantly enriched in diseased tomato treated with nanoparticles, including order *Burkholderiales* and family *Comamonadaceae* (Figure 4B).

There were 12 biomarkers with LDA scores >4 in the diseased tomato treated with thiodiazole-copper. Thiodiazole-copper significantly increased the abundance of family *Comamonadaceae*, order *Burkholderiales*, genera *Roseateles* and *Pelomonas* (LDA > 4) in healthy tomato (Figure 4C). In diseased tomato roots, order *Enterobacterales* and family *Enterobacteriaceae* were prevalent in thiodiazole-copper treatments, whereas the genera *Chujaibacter* and *Variovorax* exhibited higher numbers in nanoparticle treatments (Figure 4D). In the healthy tomato roots, the class *Alphaproteobacteria*, order *Rhizobiales*, family *Rhodanobacteraceae* and *Xanthobacteraceae*, phyla *Bacteroidota*, and 26 additional biomarkers were prevalent in the thiodiazole-copper group, while the order *Burkholderiales*, family *Comamonadaceae*, class *Gammaproteobacteria*, genera *Roseateles* and *Massilia* were more abundant in the Cu-Ag nanoparticles group (Figure 4E). The LEfSe study indicated substantial alterations in the endophytic microbe community of healthy and diseased tomato roots after treatments.

### Nanoparticle treatment enables more bacteria to participate in network interactions

**Figure 5:**
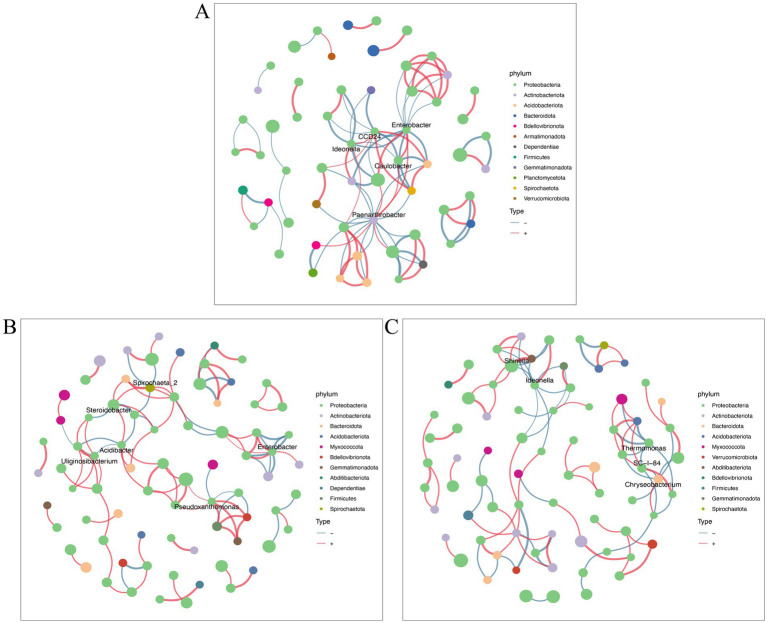
Bacterial networks of tomato roots after different treatments. Groups G6 and G9 **(A)**. Groups G6 and G7 **(B)**. Groups G6 and G8 **(C)**. Networks were constructed at the ASV level. The size of nodes (ASVs) represents the relative abundance of microbes at the phylum level. Connections are drawn between nodes where *p* < 0.01 and SpearmanCoef > 0.8. The blue lines and red lines represent negative correlation and positive correlation.

A co-occurrence network was employed to investigate the intricate relationships among endophytic microbial communities among various tomato treatment groups. *Enterobacter*, *Paenarthrobacter*, *CCD24*, *Ideonella,* and *Caulobacter* were mainly highly connected nodes in the endophytic bacterial network between control healthy and control diseased tomato roots (Figure 5A). *Enterobacter*, *Spirochaeta_2*, *Steroidobacter*, *Acidibacter*, *Uliginosibacterium*, and *Preudoxanthomonas* were the keystone taxa identified in the comparative groups of control diseased tomato and diseased tomato treated with nanoparticles (Figure 5B). The link numbers of *Shinell*, *Ideonella*, and *Thermomonas* were slightly higher in the comparison groups of control infected tomato and diseased tomato treated with thiodiazole-copper (Figure 5C). These findings demonstrated that Cu-Ag nanoparticles and thiodiazole-copper influenced the endophyte network of tomato roots, with with an increase in bacterial groups involved in the network interactions in diseased tomato treated with nanoparticles.

### Cu-Ag nanoparticles enhanced the disease resistance function of endophytic bacterial community

**Figure 6:**
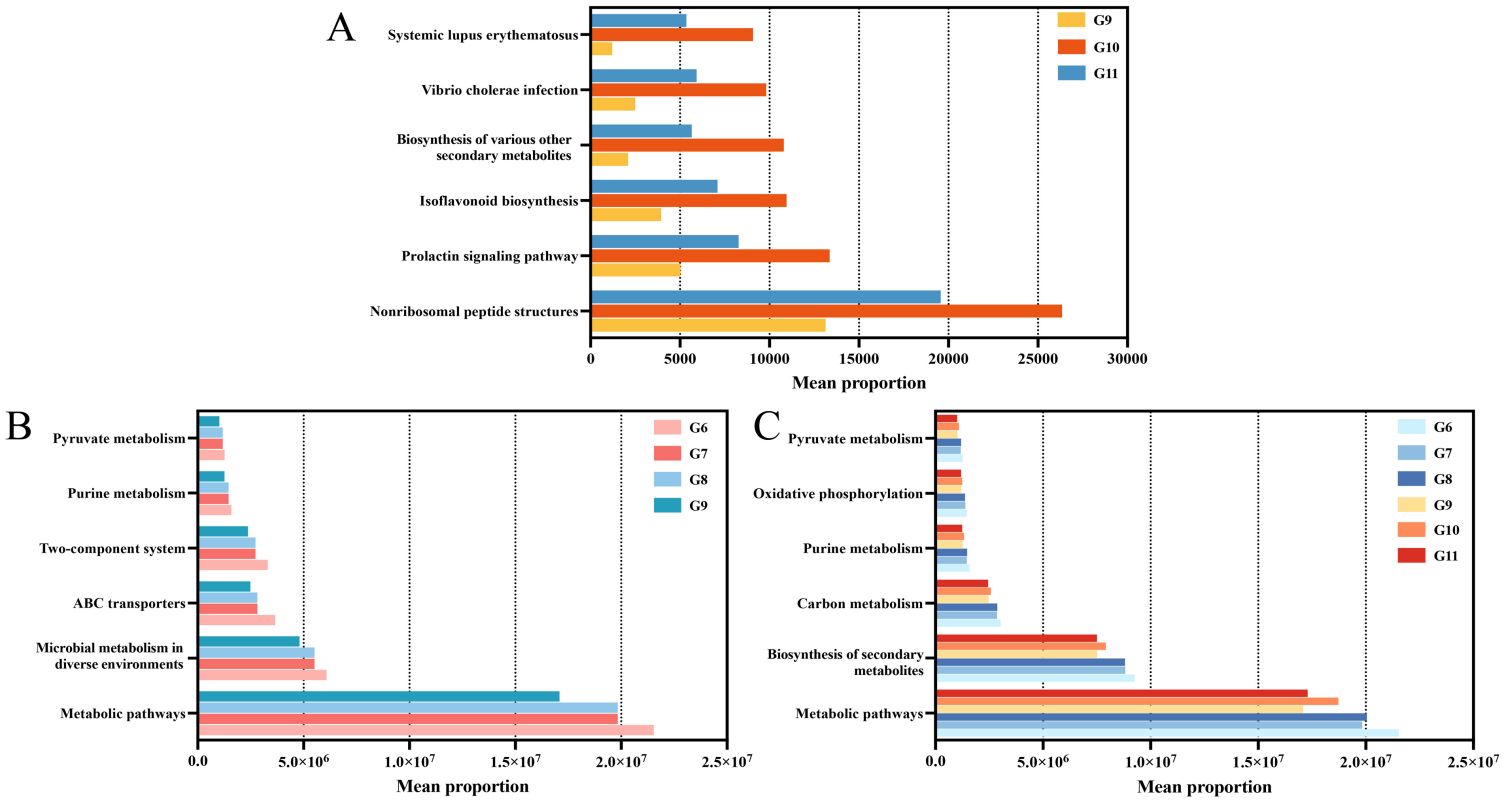
Predicting results for the significantly different metabolic pathways of endophytic microbial communities in tomato roots. Groups G9, G10, and G11 **(A)**. Groups G9, G6, G7, and G8 **(B)**. Groups G6, G7, G8, G9, G10, and G11 **(C)**.

Functional prediction was used to detect changes in bacterial community functions based on the Kyoto Encyclopedia of Genes and Genomes (KEGG) pathway database. The pathways enriched included systemic lupus erythematosus, *vibrio cholerae* infection, biosynthesis of various other secondary metabolites, isoflavonoid biosynthesis, prolactin signaling pathways, and nonribosomal peptide structures in healthy tomatoes treated with Cu-Ag nanoparticles and thiodiazole-copper. All six pathways showed the highest levels of enrichment in tomatoes treated with nanoparticles (Figure 6A). The functional predictions of endophyte communities are differ in diseased tomato. Pathways associated with pyruvate metabolism, purine metabolism, two-component systems, ABC transporters, and microbial metabolism were upregulated following the invasion of *R. solanacearum*. Notably, these pathways decreased after treatment with Cu-Ag nanoparticles and thiodiazole-copper in diseased tomato roots (Figure 6B). The pathways of pyruvate metabolism, oxidative phosphorylation, purine metabolism, carbon metabolism, production of secondary metabolites, and metabolic pathways displayed considerable variations in the tomato roots of all treatments. The biosynthesis of secondary metabolites and metabolic pathways exhibited significant alterations (Figure 6C). Functional predictions demonstrated that the metabolic pathways of the microbial community changed significantly between tomato roots treated with Cu-Ag nanoparticles and thiodiazole-copper.

To further investigate the potential effects of nanomaterials on endophytic microbial communities, a heatmap was generated to visualize the differential expression of enzymes and proteins in tomato roots. The abundance of ABC. PE. P (K02033), ABC. PE. P (K02044), methyl-accepting chemotaxis proteins (MCPs) (K03406), LacI family (K02529), and glutathione s-transferase enzymes (K00799) substantially increased in the endophyte communities of infected roots (Supplementary Figure S5A). The application of nanoparticles and thiodiazole-Cu reduced the expression of protein-glutamate methylesterase (CheB) (K03412), LacI family (K02529), GntR family transcriptional regulator (K00375), purine-binding chemotaxis protein CheW (CheW) (K03408), glycine cleavage system transcriptional activator (K03566), and Lrp/AsnC family transcriptional regulator (K03719) compared with control diseased roots (Supplementary Figure S5B). The abundance of sensor histidine kinase TctE (K07649), multiple sugar transport system ATP-binding protein (K10111), and two-component system (K02483) increased after applying nanoparticles in healthy tomato roots (Supplementary Figure S5C). In summary, exposure to Cu-Ag nanoparticles might influence the functionality of the endophytic community in the roots, potentially influencing metabolism in the roots.

### Nanoparticles influenced element contents

The Ag, Cu, P, and Mo contents in tomato roots were determined. The Ag contents in diseased (11.75 ± 0.65 mg/kg) and healthy tomato (11.95 ± 0.25 mg/kg) treated with nanoparticles were higher compared with the other treatment groups. The Cu contents in diseased (11.3 ± 0.7 mg/kg) and healthy tomato (14.3 ± 0.3 mg/kg) treated with nanoparticles were higher compared with the other treatment groups, whereas the Cu content in diseased tomato treated with nanomaterials was lower compared with healthy tomato also treated with nanomaterials. No significant difference was observed in the Cu and Ag contents between healthy control tomatoes and infected tomatoes, indicating that pathogens did not affect the Cu and Ag contents in tomato roots (Supplementary Figures S6A,B). The P content in infected tomato treated with Cu-Ag nanoparticles and thiodiazole-copper did not vary substantially from control infected tomato, and the P content in the healthy tomato group was also not significantly different (Supplementary Figure S6C). The infected tomato treated with nanoparticles (1.395 ± 0.025 mg/kg) showed more than the control infected tomato (0.9645 ± 0.095 mg/kg) and demonstrated the highest content across all samples (Supplementary Figure S6D). Nanomaterials influence the element content of tomato roots; nonetheless, the potential effects of these alterations on plant metabolism require further study.

## Discussion

The invasion of *R. solanacearum* in tomatoes has resulted in significant economic losses (Kashyap et al., 2023). Metal nanoparticles, such as CuO, ZnO, MgO, and Ag/SiO₂, have shown efficiency against tomato bacterial wilt due to their extensive surface area, simplistic binding, and multiple antibacterial mechanisms (Cai et al., 2018; Khan et al., 2021; Pham et al., 2021; Xia et al., 2024). However, the current research regarding the interactions between nanoparticles and the endophytic microbial communities of plants after exposure to nanoparticles remains limited. Microbiome analyses were conducted on healthy tomato and tomato infected with *R. solanacearum*, both treated with Cu-Ag nanoparticles and thiodiazole-copper, to improve understanding of the alterations in the endophytic bacterial composition of tomato roots triggered by nanoparticles. The efficiency of Cu-Ag nanoparticles in protecting tomatoes against tomato bacterial wilt has been shown in a previous study (Ning et al., 2024). In this study, the influence of nanoparticles on the composition of endophytic bacteria in diseased tomato roots was considered by employing functional prediction to describe possible metabolic alterations.

The variety of microbial communities plays a vital role in maintaining the integrity of the soil ecosystem and sustaining plant ecological functions (Beattie et al., 2024). Previous reports indicate that certain plant diseases have resulted in a decrease in the diversity of host endophytic microbial communities, such as wilt disease in pine and bacterial blight disease in rice (Li D. et al., 2024; Yang et al., 2020). The PCoA and NMDS plots showed that samples from the same treatment group tended to cluster together; in contrast, samples from different treatments were spread apart, indicating a significant disparity in the composition of the endophytic microbial community in the healthy and diseased tomato roots, both treated with Cu-Ag nanoparticles and thiodiazole-copper.

A plethora of studies demonstrate that pathogen invasion may influence the host’s microbial community; therefore, the endophytic composition of tomato roots was analyzed under various treatments. At the phylum level, Cu-Ag nanoparticles substantially increased the relative abundances of *Actinobacteriota* and *Myxococcota* in the diseased tomato roots. Research has shown the significance of *Actinobacteriota* and *Myxococcota* regarding their ability to withstand various environmental stresses (Li Y. et al., 2024). *Actinobacteriota* generates active enzymes that efficiently degrade organic carbon, thus enhancing the decomposition of additional organic matter, while *Myxococcota* has shown the capacity to improve maize yield (Yu L. et al., 2024). Meanwhile, Cu-Ag nanoparticles augmented the number of *Acidobacteriota* and *Firmicutes* in diseased tomato roots. *Acidobacteria* could enhance soil quality, promote ginger growth, and produce antibacterial substances that prevent pathogens from adhering to plant roots, thus reducing the incidence of diseases transmitted via soil (Lee et al., 2021). *Firmicutes* participate in carbon source utilization and can degrade cellulose, lignin, and wood fibers by producing hydrolytic enzymes (Simpson and Campbell, 2015). Furthermore, research has demonstrated that the incidence of tomato bacterial wilt increases when communities of *Actinobacteria* and *Firmicutes* are particularly damaged (Wang et al., 2023). Our data show that the invasion of *R. solanacearum* decreased the relative abundances of *Actinobacteriota.* Notably, the observed increase in *Actinobacteriota, Myxococcota, Acidobacteriota,* and *Firmicutes* in root samples aligns with the hypothesis that nanoparticles may decrease pathogenic bacterial populations while promoting the growth of certain beneficial bacterial taxa (Meena et al., 2021).

*R. solanacearum* is a Gram-negative, plant-pathogenic bacterial species classified in the family *Burkholderiaceae*, order *Burkholderiales*, and class *Betaproteobacteria* (Paudel et al., 2020). The invasion of *R. solanacearum* triggered to a *Burkholderiales* abundance of 23.77% in the control infected tomato roots, while the application of Cu-Ag nanoparticles reduced the abundance to 12.58%. At the family level, Cu-Ag nanoparticles reduced the abundance of *Burkholderiaceae* in diseased tomato roots. The study revealed a significant alteration in the *Burkholderiales* following the application of AgNPs to the soil; additionally, research demonstrated that CuO₂, ZnO₂, and FeO₂ decreased the abundances of *Intrasporangiaceae*, *Nocardioidaceae*, and *Burkholderiaceae* within the rhizosphere bacterial community of tomatoes affected by *R. solanacearum* at the second week (Zhang et al., 2021; Jiang et al., 2021). In the future, further studies can be conducted to investigate the processes of endophytic microbial community restructuring in tomato under pathogen invasion and nanoparticle exposure.

The influence of Cu-Ag nanoparticles on tomato was investigated by analyzing the potential ecological functions of endophytic microbial communities. The plant microbiome is known as the second genome of plants, and microbial communities can modulate certain functional genes associated with protein transport systems, cellular detoxification, and protection of the hosts from oxidative damage (Kassogue et al., 2020; Yu W. et al., 2024). The LacI family functions as genetic regulatory proteins and sugar effectors, efficient in regulating carbohydrate utilization and controlling metabolic genes (Ravcheev et al., 2014; Swint-Kruse and Matthews, 2009). MCPs exhibit important functions in cell survival, pathogenicity, biodegradation, and the cycling of carbon, nitrogen, and sulfur, as well as signal detection and cellular responses. Notably, MCPs play a vital role in bacterial chemotaxis, as well as the processes of symbiosis and pathogenesis (Hida et al., 2020; O'Neal et al., 2019; Salah Ud-Din and Roujeinikova, 2017). The functional prediction results indicate that the LacI family and MCPs were elevated after the invasion of *R. solanacearum*. In contrast, applying nanoparticles to diseased tomato plants caused a reduction in the expression of the LacI family.

Metal ions markedly reduced the adhesive capacity of *vibrio harveyi*, whereas CheB regulates the bacteria’s abilities involving adhesion, motility, chemotaxis, and biofilm formation (Xu et al., 2020). The GntR family protein regulators are prevalent in bacteria, playing roles in metabolic processes, glucose metabolism, bacterial pathogenicity, and virulence (Jiang et al., 2024; Wang et al., 2020). CheW response regulators can transmit signals and coordinate chemoreceptors to sense various attractants and repellents (Arapov et al., 2020; Kennedy et al., 2022). The glycine cleavage system inhibits ammonia production, potentially enhancing the efficacy of conventional antibiotics (Avalos et al., 2020). The H protein is the target of oxadiazole sulfones in *R. solanacearum* with broad-spectrum biological activity, including antibacterial, antifungal, and antiviral activities (Chen et al., 2020). The Lrp/AsnC transcriptional regulator family is widespread in bacteria and is associated with regulating amino acid metabolism, stress tolerance, virulence, and antibiotic resistance (Feng et al., 2024; Matarredona et al., 2023). Moreover, exogenous substances could affect the expression levels of these genes. Functional prediction data reveal that Cu-Ag nanoparticle-treated diseased tomato had lower expression levels of these genes compared with the control diseased tomato, indicating that the diverse impacts of nanomaterials on tomatoes.

The sensor histidine kinase TctE, ATP-binding proteins, and two-component systems play an essential role in the carbon metabolism, tricarboxylic acids, biofilm formation, and desiccation tolerance (Evans et al., 2020; Guo et al., 2024; Ji et al., 2024; Taylor et al., 2019). The expression levels of these genes increased in the healthy tomato roots treated with nanoparticles, implying that adding nanoparticles does not interfere with normal physiological functions in healthy tomato. Functional predictions show that Cu-Ag nanoparticles may influence the pyruvate metabolism, oxidative phosphorylation, purine metabolism, carbon metabolism, secondary metabolite synthesis, and metabolic pathways in endophytic microbial communities. Current studies on the influence of nanoparticles on tomato lack adequate depth. Future research could investigate the dynamic alterations in endophytic microbial communities of tomato plants under various treatments. Functional predictions suggest that Cu-Ag nanoparticles may affect metabolic pathways; the metabolomic analysis might be utilized to investigate the interactions between tomatoes and Cu-Ag nanoparticles in further research.

## Conclusion

This study investigated the influence of Cu-Ag nanoparticles on the microbiota of tomatoes infected with *R. solanacearum*. The application of nanoparticles caused alterations in the endophytic bacteria of the infected tomato roots. Nanoparticles influenced the endophytic microbial profiles of the tomato roots by enhancing the relative abundance levels of beneficial microorganisms, promoting an increase in the diversity of groups participating in the bacterial interaction network. Moreover, functional predictions indicate that Cu-Ag nanoparticles may influence the metabolic pathways of endophytic microbial populations in the roots of infected tomatoes. These findings will enhance comprehension of the influence of nanoparticles on endophytic microbial communities and the interactions between endophytes and nanoparticles, thus providing a basis for applying nanoparticles in agricultural green prevention and control strategies for soil-borne diseases.

## Data Availability

The datasets presented in this study can be found in online repositories. The names of the repository/repositories and accession number(s) can be found in the article/Supplementary material.
