# Supplementary material for: Cu-Ag nanoparticles positively modulating the endophytic bacterial community in tomato roots affected by bacterial wilt
Source: Front Microbiol. 2025 Jul 16;16:1579517. doi: 10.3389/fmicb.2025.1579517 (PMC12307443; doi:10.3389/fmicb.2025.1579517)
Supplement: Supplementary file 1 [file Data_Sheet_1.docx]

Supplementary Material

**Cu-Ag Nanoparticles Positively Modulating the Endophytic Bacterial Community in Tomato Roots Affected by Bacterial Wilt**


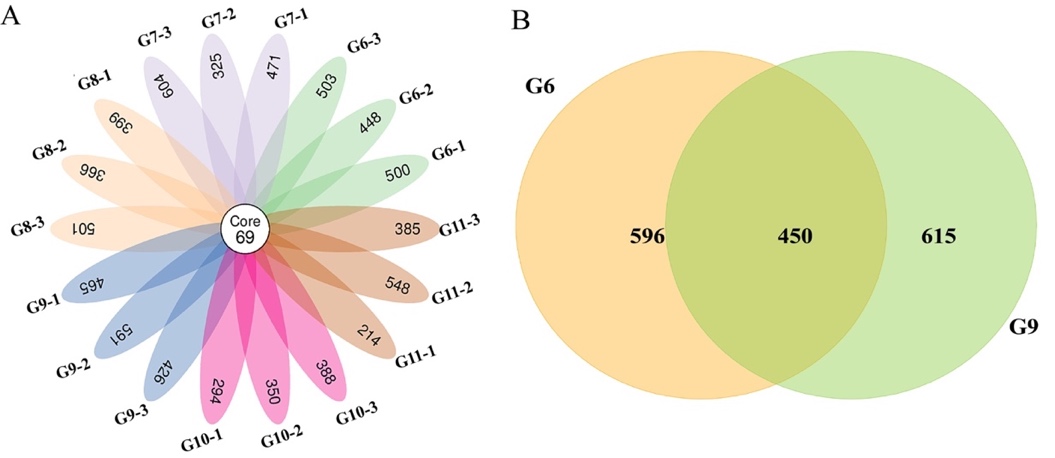


**Supplementary Figure 1.** Venn diagram illustrating the relationships between shared and unique ASVs in all roots sample (A). Groups G6 and G9 (B).


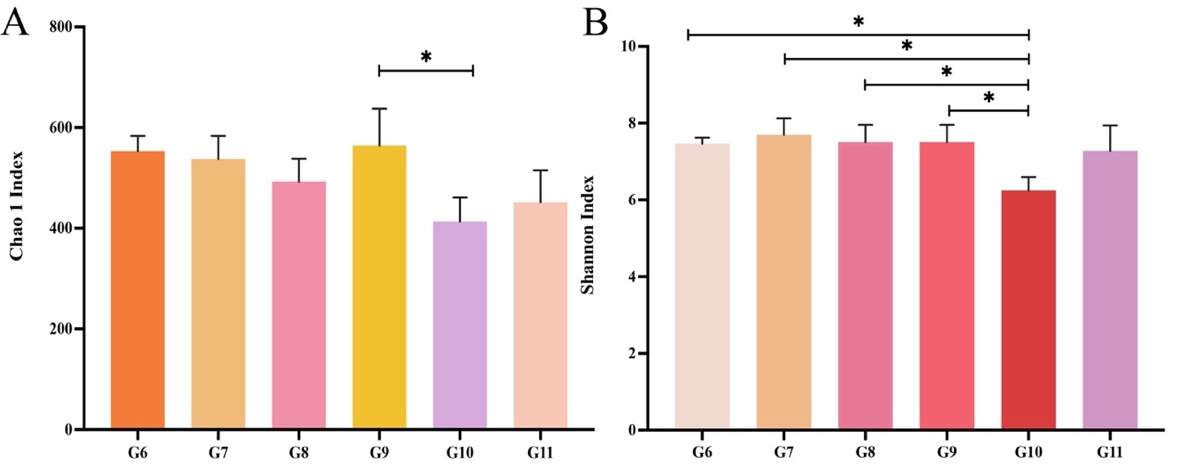


**Supplementary Figure 2.** The alpha-diversity of the endophytic bacterial community in all tomato roots. Chao1 estimator (A). Shannon index (B). Statistical significance was determined based on Tukey’s HSD test. * p < 0.05.

**
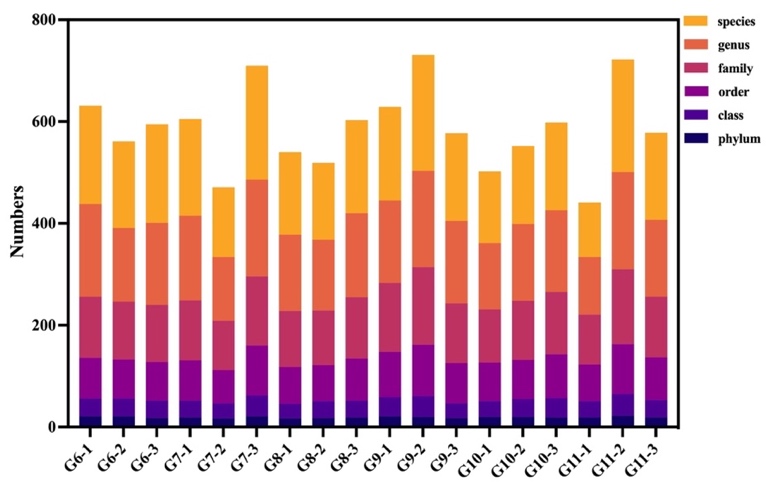
**

**Supplementary Figure 3.** Taxonomic compositions at annotation level in each sample.


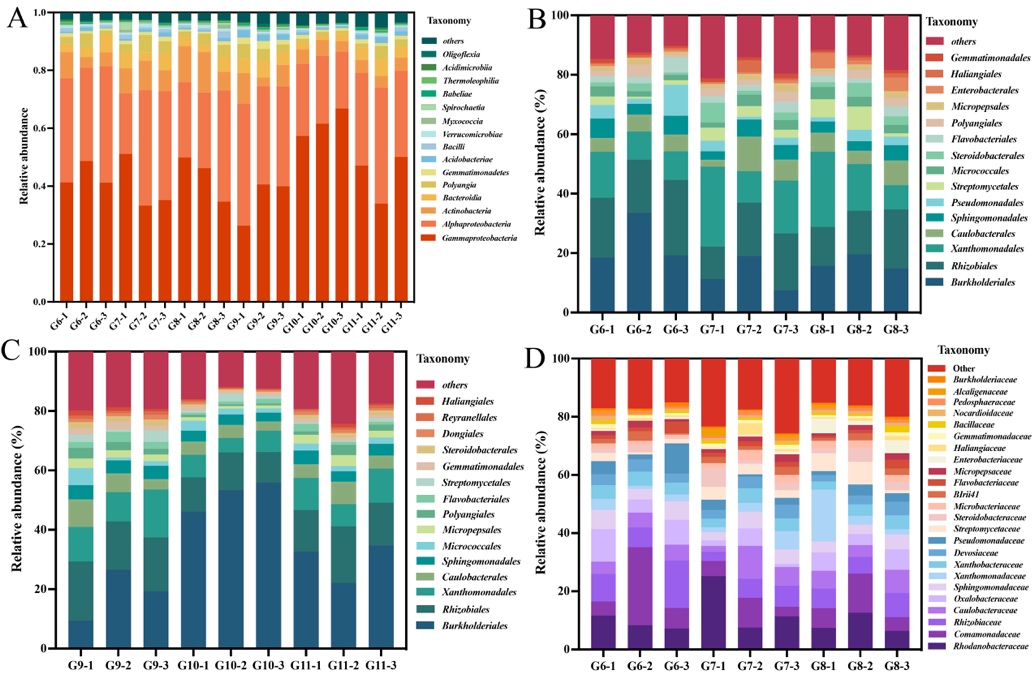


**Supplementary Figure 4.** Stacked bar graph for the relative abundance of bacterial 16S rRNA. Top 15 relative abundances of bacterial taxa at the class level of all samples (A). The bacterial community abundance at order levels of infected tomato plants (B) and healthy tomato (C) treated with Cu-Ag nanoparticles and chemical pesticide. The bacterial community abundance at family levels of infected tomato plants (D) treated with Cu-Ag nanoparticles and chemical pesticide.


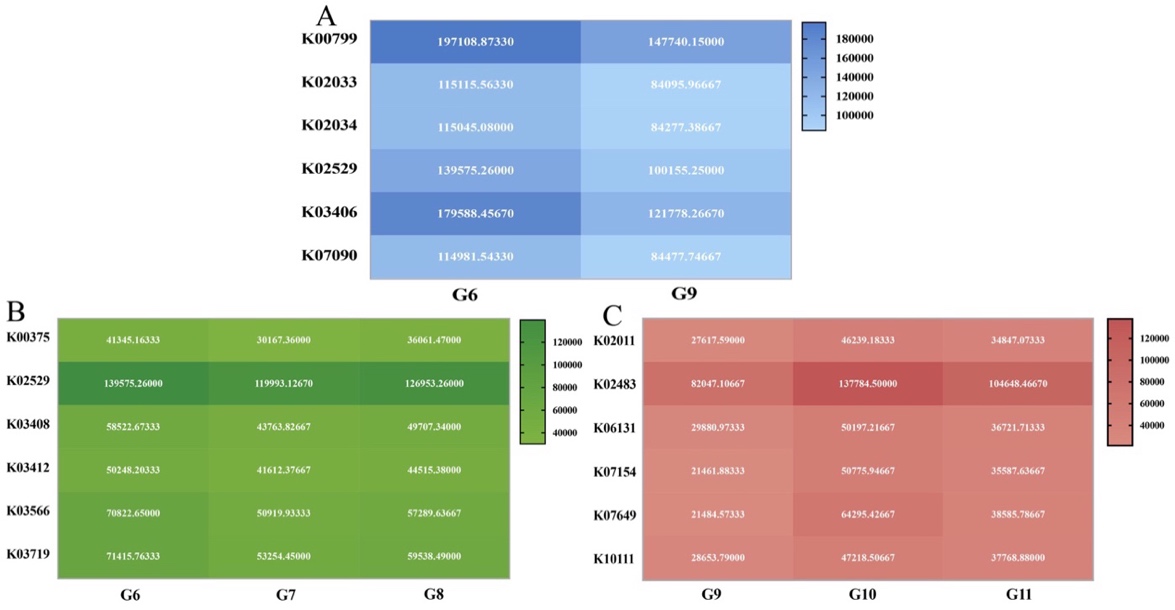


**Supplementary Figure 5.** Heatmap of predicting the gene function of endophytes with PICRUSt2 analysis. Groups G6 and G9 (A). Groups G6, G7, and G8 (B). Groups G9, G10, and G11 (C).


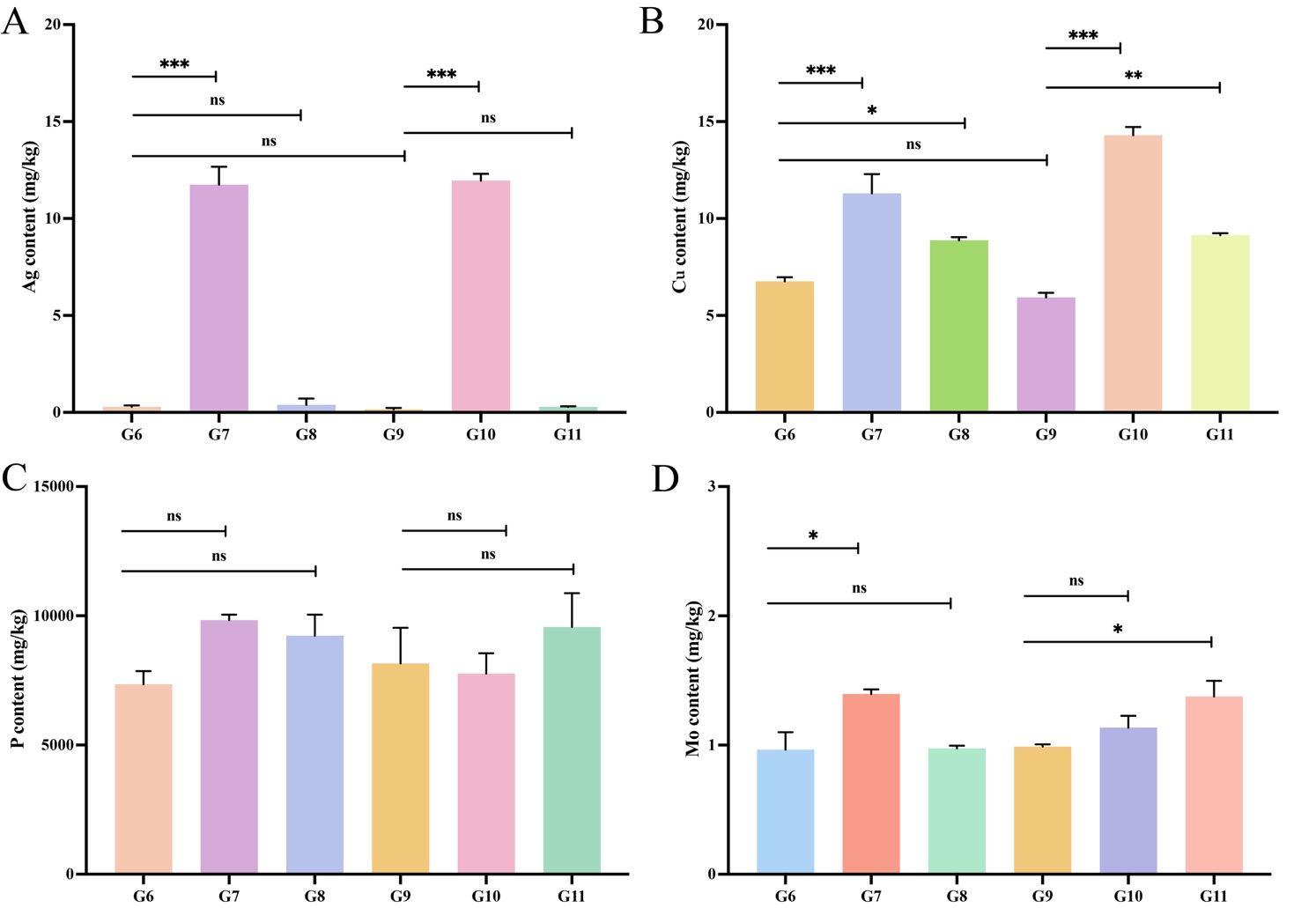


**Supplementary Figure 6.** The Ag (A), Cu (B), P (C), and Mo (D) content in roots of healthy and infected tomato after treatment with of bimetallic Cu-Ag nanoparticles or chemical pesticide. Data are mean ± SD (standard deviation); one-way ANOVA with Duncan’s test. *P < 0.05, **P < 0.01, ***P< 0.001, and ns indicates no significant difference.
